# Apolipoprotein E gene and age-related macular degeneration in a Chinese population

**Published:** 2011-04-20

**Authors:** Erdan Sun, Apiradee Lim, Xipu Liu, Torkel Snellingen, Ningli Wang, Ningpu Liu

**Affiliations:** 1 Beijing Tongren Eye Center, Beijing Tongren Hospital, Capital Medical University, Beijing Ophthalmology and Visual Sciences Key Laboratory, Beijing, China; 2 Department of Mathematics and Computer Science, Faculty of Science and Technology, Prince of Songkla University, Pattani Campus, Pattani, Thailand; 3 Sekwa Eye Hospital, Beijing, China

## Abstract

**Purpose:**

To examine the association between apolipoprotein E (*APOE*) polymorphisms and age-related macular degeneration (AMD) in a Chinese population.

**Methods:**

The study consisted of 712 subjects, including 201 controls, 363 cases with early AMD, and 148 cases with exudative AMD. Genomic DNA was extracted from venous blood leukocytes. Common allelic variants of *APOE* (ε2, ε3, and ε4) were analyzed by PCR and direct sequencing.

**Results:**

*APOE* ε3ε3 was the most frequent genotype, with a frequency of 72.6% in controls, 72.5% in early AMD, and 70.3% in exudative AMD. Frequency of the ε2 allele was 6.7% in controls, 7.4% in early AMD, and 8.8% in exudative AMD. Frequency of the ε4 allele was 8.7% in controls, 7.7% in early AMD, and 7.8% in exudative AMD. No statistically significant difference in APOE genotype and allele frequency distribution was observed among controls, cases with early AMD, and cases with exudative AMD. For ε2 allele carriers, the odds ratio was 1.12 (95% confidence interval [CI], 0.65–1.93) for early AMD and 1.06 (95% CI, 0.53–2.10) for exudative AMD. For ε4 allele carriers, the odds ratio was 1.04 (95% CI, 0.61–1.75) for early AMD and 0.83 (95% CI, 0.42–1.62) for exudative AMD.

**Conclusions:**

Our data provide no evidence to support an association of *APOE* polymorphisms with early or exudative AMD, suggesting that *APOE* is less likely to be a major AMD susceptibility gene in the Chinese population.

## Introduction

Age-related macular degeneration (AMD) causes irreversible central vision loss and is the leading cause of legal blindness in the elderly population. Evidence suggests that AMD is a complex disease caused by actions and interactions of multiple genes and environmental factors [1-10]. Apolipoprotein E is a transport protein of lipid and cholesterol in the nervous system, playing a pivotal role in the re-innervation process following nervous system injury [11-14]. The gene for apolipoprotein E (*APOE*), located on chromosome 19q13.2, is polymorphic, with three major allelic variants, ε2, ε3, and ε4 [12,15]. The *APOE* ε4 allele has been associated with a variety of neurodegenerative and cardiovascular diseases, most notably Alzheimer disease. Interestingly, APOE is also a ubiquitous component of drusen [16-21], a hallmark of early AMD. Various clinical signs of AMD are also mimicked in *APOE* knockout [20] or transgenic animal models [21], suggesting that *APOE* may play a role in the pathogenesis of AMD. Several genetic association studies have shown that the *APOE* ε4 allele is associated with a protective effect against AMD [22-28], whereas the ε2 allele may be weakly associated with an increased risk [16,27-31]. Other studies, however, do not support this association between *APOE* and AMD [32-37]. Given the inconsistencies of the previous studies, it was imperative to investigate this association further. The purpose of this study was to verify the possible association of *APOE* polymorphisms with early or late AMD in an independent cohort of a Chinese population.

## Methods

### Subjects and clinical evaluation

This case-control cohort included 201 control subjects, 363 cases with early AMD, and 148 cases with exudative AMD. Gender and age breakdown of the controls and cases were given in Table 1. The study participants were recruited through a community-based eye disease screening program in urban Beijing and outpatients visiting the Beijing Tongren Hospital. Subjects aged 50 years or older with or without signs of early or late AMD were included in the study. Exclusion criteria included any contraindications to pupil dilation, advanced cataract or other conditions that impeded a clear view of the retina, or patients with eye diseases, such as glaucoma, uveitis, and chorioretinal diseases, other than AMD. Patients with diabetes were also excluded. The study protocol was reviewed and approved by the Ethics Committee of the Beijing Tongren Hospital. Informed consent was obtained from all participants, and the procedures used conformed to the tenets of the Declaration of Helsinki for research involving human subjects.

**Table 1:** Characteristics of study subjects.

		**Cases with AMD**	
**Age (years)**	**Controls (n=201)**	**Early AMD (n=363)**	**Exudative AMD (n=148)**
mean±SD	63.7±7.6	67.2±7.5	65.6±8.6
Range	50 - 84	50 - 84	50 - 89
**Age group**			
50–54	21 (10.4%)	20 (5.5%)	14 (9.5%)
55–59	52 (25.9%)	37 (10.2%)	27 (18.2%)
60–64	50 (24.9%)	82 (22.6%)	24 (16.2%)
65–69	23 (11.4%)	77 (21.2%)	39 (26.4%)
≥70	55 (27.4%)	147 (40.5%)	44 (29.7%)
**Gender**			
Female	138 (68.7%)	233 (64.2%)	56 (37.8%)
Male	63 (31.3%)	130 (35.8%)	92 (62.2%)
**Cigarette Smoking**			
Never	153 (76.1%)	284 (78.2%)	80 (54.1%)
Past	24 (11.9%)	44 (12.1%)	31 (20.9%)
Current	24 (11.9%)	35 (9.6%)	37 (25.0%)

This table summarizes the general characteristics of study subjects with or without age-related macular degeneration (AMD). SD represents standard deviation.

**Table 2:** Apolipoprotein E (APOE) genotype and allele frequency distribution among study subjects.

		**Cases with AMD**	
**Genotype**	**Controls**	**Early AMD**	**Exudative AMD**
ε2ε2	0 (0)	2 (0.6%)	1 (0.7%)
ε2ε3	24 (11.9)	46 (12.7%)	20 (13.5%)
ε2ε4	3 (1.5%)	4 (1.1%)	4 (2.7%)
ε3ε3	146 (72.6%)	263 (72.5%)	104 (70.3%)
ε3ε4	24 (11.9%)	44 (12.1%)	19 (12.8%)
ε4ε4	4 (2.0%)	4 (1.1%)	0 (0)
Total	201 (100%)	363 (100%)	148 (100%)
P-value	—	0.885	0.416
**H-W**			
χ^2^	6.265	1.846	2.994
p	0.099	0.605	0.393
**Allele**			
ε2	27 (6.7%)	54 (7.4%)	26 (8.8%)
ε3	340 (84.6%)	616 (84.8%)	247 (83.4)
ε4	35 (8.7%)	56 (7.7%)	23 (7.8%)
Total	402 (100%)	726 (100%)	296 (100%)
p-value	—	0.776	0.558
**Carrier**			
ε*2	24 (12.1%)	48 (13.4%)	21 (14.6%)
ε3ε3	146 (73.7%)	263 (73.3%)	104 (72.2%)
ε*4	28 (14.1%)	48 (13.4%)	19 (13.2%)
Total	198 (100%)	359 (100%)	144 (100%)
p-value	—	0.899	0.793

The genotype and allele frequency distributions in controls and cases with early or exudative age-related macular degeneration (AMD) are summerized in this table. Data are expressed as number (%). For carriers, the ε*2 group includes genotypes of ε2ε2 and ε2ε3, whereas the ε*4 group includes genotypes of ε3ε4 and ε4ε4. Subjects with ε2ε4 genotype (3 in controls, 4 in early AMD, 4 in exudative AMD) are excluded from either ε*2 or ε*4 group. P-value represents comparison of genotype or allele frequency distribution between cases and controls. H-W represents Hardy–Weinberg equilibrium.

All subjects underwent a comprehensive ophthalmological examination, including visual acuity measurement, slit-lamp biomicroscopy, and detailed fundus examination after pupil dilation, using 0.5% tropicamide and 5% phenylephrine (Santen Pharmaceutical, Osaka, Japan), performed by a retinal specialist. Smoking history was ascertained using a standard questionnaire. Stereoscopic mydriatic 30° color fundus photographs were taken from both eyes, using a digital fundus camera (Zeiss Oberkochen, Oberkochen, Germany) centered on the fovea. Fundus photographs were assessed by two independent readers, and a retinal specialist was used as a third person to make final judgment in case of any discrepancies. Early AMD was defined as the presence of soft drusen (>63 µm) with or without pigment abnormalities [24]. Individuals with only hard drusen (classified as drusen <63 µm in size) or with only pigmentary changes were not classified as having AMD as it was imperative to be able to classify individuals as positive for disease with certainty. Individuals were selected as control subjects based on the presence of normal fundus or having only a few (less than ten) hard drusen in both eyes [24,38]. Patients with late AMD were examined and confirmed by fluorescein and/or indocyanine green fundus angiography.

### Apolipoprotein E genotyping

Blood samples were collected from all participants and stored at −80 °C before DNA extraction. Genomic DNA was isolated from venous blood leukocytes using a genomic DNA extraction and purification kit (TIANamp Swab DNA Kit; Tiangen Biotech, Beijing, China) according to the manufacturer’s protocol. Briefly, lysis buffer was added to blood samples, mixed, and the tubes centrifuged. The supernatant was discarded and pellet re-suspended in a solution of proteinase K and sodium dodecyl sulfate. The mixture was incubated at 65 °C for 30 min. Genomic DNA was precipitated by isopropanol, rinsed with ethanol, and finally re-suspended in TE buffer (1M Tris-HCl; 0.5M EDTA; pH 8.0). PCR amplification was undertaken in a GeneAmp PCR 9700 thermal cycler (Applied Biosystems, Foster City, CA). A 241-bp PCR product encompassing amino acid position 112 was produced by using the forward primer 5′-GCC TCC CAC TGT GCG A-3′ and the reverse primer 5′-GGC CGA GCA TGG CCT G-3′. A 150-bp product encompassing amino acid position 158 was produced by using the forward primer 5′-ACC GAG GAG CTG CGG G-3′ and the reverse primer 5′-CTC GCG GAT GGC GCT GA-3′. PCR conditions used for amplification were 94 °C for 5 min, followed by 40 cycles of 94 °C for 30 s, 65 °C for 30 s, and 72 °C for 30 s. The final extension step at 72 °C was lengthened to 10 min. PCR products were then directly sequenced using an automatic ABI 3730XL DNA Analyzer (Applied Biosystems). Single nucleotide polymorphisms (SNPs) at amino acid position 112 (C112R or c.388T>C of GenBank reference sequence NM000041.2) and 158 (R158C or c.526C>T of reference sequence NM000041.2) were then used to determine the *APOE* allelic variants ε2 (112C, 158C), ε3 (112C, 158R, reference sequence NM000041.2), and ε4 (112R, 158R).

### Statistical analysis

The data were analyzed using the R statistical analysis package. Hardy–Weinberg equilibrium was assessed using the chi-square test comparing observed with expected proportions. Distributions of *APOE* genotypes (ε2ε2, ε2ε3, ε2ε4, ε3ε3, ε3ε4, ε4ε4) and alleles (ε2, ε3, ε4) were analyzed using the χ^2^ test and Fisher’s exact test when the expected frequency was <5. We defined carriers of the ε2 allele to include genotypes ε2ε2 and ε2ε3 and carriers of the ε4 allele to include genotypes ε3ε4 and ε4ε4. Individuals with the ε2ε4 genotype were excluded from all analyses to avoid potential confounding effects. A logistic regression model was used to calculate the odds ratio (OR) and 95% confidence interval (CI) of early or late AMD, comparing ε2 or ε4 allele carriers to the ε3ε3 genotype as the reference, adjusted for age, gender, and cigarette smoking. A risk score model was used to evaluate the variation in *APOE* polymorphisms, as suggested in the literature [37]. Briefly, the model respectively assigned +1, 0, or −1 per ε2, ε3, or ε4 allele. As a result, the genotypes of ε2ε2, ε2ε3, ε2ε4, ε3ε3, ε3ε4, and ε4ε4 will respectively have scores of +2, +1, 0, 0, −1, and −2. The statistically significant level was set at p<0.05.

## Results

A total of 712 subjects participated in the study, including 201 control subjects, 363 cases with early AMD in one or both eyes, and 148 cases with exudative AMD in at least one eye. Fluorescein angiography was undertaken in all cases with exudative AMD, and indocyanine green angiography was available in 42 cases. No atrophic AMD patient was identified among the study participants. The general characteristics of the study subjects are given in Table 1.

The *APOE* genotype and allele frequency distribution among study subjects are given in Table 2. Frequencies of *APOE* genotypes were under Hardy–Weinberg equilibrium in controls and cases with early or exudative AMD (p≥0.099). The ε3ε3 was the most frequent genotype with a frequency of 72.6% in controls, 72.5% in early AMD, and 70.3% in exudative AMD. Frequency of the ε2 allele was 6.7% in controls, 7.4% in early AMD, and 8.8% in exudative AMD. Frequency of the ε4 allele was 8.7% in controls, 7.7% in early AMD, and 7.8% in exudative AMD. No statistically significant differences between controls and early AMD or between controls and exudative AMD were found for any genotype or allele frequencies.

The OR and risk scores of *APOE* alleles associated with early or exudative AMD are given in Table 3. After adjustment for age, gender, and cigarette smoking, the OR for ε2 allele carriers was 1.12 (95% CI, 0.65–1.93) for early AMD and 1.06 (95% CI, 0.53–2.10) for exudative AMD. For ε4 allele carriers, the OR was 1.04 (95% CI, 0.61–1.75) for early AMD and 0.83 (95% CI, 0.42–1.62) for exudative AMD. No association between increasing *APOE* risk score and early or exudative AMD was identified.

**Table 3:** Association between apolipoprotein E (*APOE*) and risk of age-related macular degeneration (AMD).

**AMD**	***APOE***	**OR (95% CI)**	
		**Crude**	**Adjusted**
Early AMD	ε2 carrier	1.11 (0.65 - 1.89)	1.12 (0.65 - 1.93)
	ε4 carrier	0.95 (0.57 - 1.58)	1.04 (0.61 - 1.75)
	*APOE* risk score	1.12 (0.82 - 1.52)	1.08 (0.79 - 1.48)
Exudative AMD	ε2 carrier	1.23 (0.65 - 2.32)	1.06 (0.53 - 2.10)
	ε4 carrier	0.95 (0.51 – 1.80)	0.83 (0.42 – 1.62)
	*APOE* risk score	1.22 (0.83 - 1.79)	1.22 (0.81 - 1.83)

Logistic regression model was used to calculate odds ratio (OR) and 95% confidence interval (CI) for early or late AMD, comparing ε2 or ε4 allele carriers vs ε3ε3 genotype as reference. The ε2 carrier includes genotypes of ε2ε2 and ε2ε3, whereas the ε4 carrier includes genotypes of ε3ε4 and ε4ε4. Subjects with ε2ε4 genotype (3 in controls, 4 in early AMD, 4 in exudative AMD) are excluded from either ε2 or ε4 carrier. *APOE* risk scores were evaluated by respectively assigning +1, 0, or −1 per ε2, ε3, or ε4 allele. As a result, genotypes of ε2ε2, ε2ε3, ε2ε4, ε3ε3, ε3ε4, and ε4ε4 would have scores of +2, +1, 0, 0, −1, and −2, respectively. Data are expressed as OR (95%CI). Adjusted OR represents data after adjustment for age, gender and cigarette smoking.

The power to detect a significant association between the *APOE* ε4 or ε2 allele and any AMD was calculated under the condition of different hypothetical OR values. To detect a significantly protective effect of the ε4 allele, the sample size in this study had 83.3% power if the ε4 allele carriers had an OR of 0.5 and 30.3% power if the ε4 allele carriers had an OR of 0.7. To detect a significant risk effect of the ε2 allele, the sample size in this study had 78.0% power if the ε2 allele carriers had an OR of 2.3 and 26.8% power if the ε2 allele carriers had an OR of 1.5.

## Discussion

The possible relationship between *APOE* polymorphisms and AMD has been examined in previous studies, most of which have focused on late AMD in Caucasian populations. In general, many of these previous studies suggest that the *APOE* ε4 allele is associated with a lower risk of late AMD [22-28], whereas less consistently the ε2 allele seems to confer an increased risk [16,27-31]. This association between *APOE* and AMD, however, is not always reproducible. Several other studies reported no evidence to support a significant association between *APOE* and late AMD in Caucasian [32-34], Hong Kong Chinese [35], and Japanese [36] populations. Moreover, no evidence of association between *APOE* and early AMD was found in a large cohort of a population-based cross-sectional study in a Caucasian population [37]. In agreement with a previous report in Hong Kong Chinese [35], our data provide no evidence to support a significant association between *APOE* and exudative AMD. In addition, no association between *APOE* and early AMD was found in this study. In a previous report in Japanese patients, *APOE* was not associated with polypoidal choroidal vasculopathy (PCV) or typical AMD [36]. In this current study, however, we were not able to differentiate PCV from typical AMD as the confirmation of PCV is solely based on indocyanine green angiography, which is not available to the majority of our patients with exudative AMD.

The lack of a significant association of *APOE* polymorphisms with AMD in the current study may be explained by the small or nonexistent effect of *APOE* on AMD in the Chinese population. It should be noted, however, that the *APOE* ε4 allele occurs less frequently in Chinese than in Caucasian control populations, making it more difficult to detect the effect of *APOE* in Chinese individuals. The frequency of the ε4 allele was reported to be 12.4%–17.9% in Caucasian control populations [16,24,25,31,33,34]. In the current study, the frequency of ε4 allele was 8.7% in controls, effectively decreasing the power of our study to replicate the findings reported in Caucasian populations. The sample size in this current study had sufficient statistical power to detect only a strong association between *APOE* and AMD. We therefore cannot exclude the possibility of a weak association between *APOE* polymorphisms and AMD in the Chinese population.

In summary, we provide no evidence to support a significant association of *APOE* polymorphisms with early or exudative AMD in this study, suggesting that *APOE* is less likely to be a major susceptibility gene to AMD in the Chinese population. Whether there is a weak association between *APOE* and AMD in Chinese, however, needs to be confirmed with a much larger sample size in future studies.
